# mRNA and miRNA Transcriptome Profiling of Granulosa and Theca Layers From Geese Ovarian Follicles Reveals the Crucial Pathways and Interaction Networks for Regulation of Follicle Selection

**DOI:** 10.3389/fgene.2019.00988

**Published:** 2019-10-23

**Authors:** Qin Li, Shenqiang Hu, Yushi Wang, Yan Deng, Shuang Yang, Jiwei Hu, Liang Li, Jiwen Wang

**Affiliations:** ^1^Farm Animal Genetic Resources Exploration and Innovation Key Laboratory of Sichuan Province, Sichuan Agricultural University, Chengdu, China; ^2^Poultry Science Institute, Chongqing Academy of Animal Science, Chongqing, China

**Keywords:** *Anser cygnoides*, follicle selection, granulosa layer, theca layer, transcriptome profiling, junctional adhesion, lipid metabolism

## Abstract

Follicle development is characterized by the recruitment, growth, selection, and dominance of follicles, and follicle selection determines the lifetime reproductive performance. However, in birds, the molecular mechanisms underlying follicle selection still remain elusive. This study analyzed genome-wide changes in the mRNA and miRNA expression profiles in both the granulosa and theca layers of geese ovarian follicles before selection (4–6- and 8–10-mm follicles) and after selection (F5). The sequencing results showed that a higher number of both differentially expressed (DE) mRNAs and DE miRNAs were identified between 8–10-mm and F5 follicles compared with those between the 4–6- and 8–10-mm follicles, especially in the granulosa layer. Moreover, a Short Time-series Expression Miner analysis identified a large number of DE mRNAs and DE miRNAs that are associated with follicle selection. The functional enrichment analysis showed that DE genes in the granulosa layer during follicle selection were mainly enriched in five pathways related to junctional adhesion and two pathways associated with lipid metabolism. Additionally, an interaction network was constructed to visualize interactions among protein-coding genes, which identified 53 junctional adhesion- and 15 lipid regulation-related protein-coding genes. Then, a co-expression network between mRNAs and miRNAs in relation to junctional adhesion was also visualized and mainly included *acy*-miR-2954, *acy*-miR-218, *acy*-miR-2970, *acy*-miR-100, *acy*-miR-1329, *acy*-miR-199, *acy*-miR-425, *acy*-miR-181, and *acy*-miR-147. Furthermore, miRNA–mRNA interaction pairs related to lipid regulation were constructed including *acy*-miR-107, *acy*-miR-138, *acy*-miR-130, *acy*-miR-128, and *acy*-miR-101 during follicular selection. In summary, these data highlight the key roles of junctional adhesion and lipid metabolism during follicular selection and contribute to a better understanding of the mechanisms underlying follicle selection in birds.

## Introduction

The annual egg production of most domestic geese breeds is approximately 20–40, which is far less compared with that of other poultry such as chickens and ducks (>300) (Buckland and Guy, 2002). This comparatively low egg production performance constitutes a substantial hindrance for the development of the goose industry. In birds, the number of produced eggs depends on the maturation of ovarian follicles, which is a complex process that consists of follicular recruitment, growth, selection, and dominance (Johnson, 2015a; Zhao et al., 2017). In laying hens, follicle selection is defined as the process through which a single follicle within a cohort of small yellow follicles (SYF; measuring 6–8 mm in diameter) is selected into the preovulatory hierarchy (Johnson and Woods, 2009; Johnson, 2015b). After selection, the follicle undergoes a rapid growth phase during which both the cell number and the surface area increase and large amounts of yolk precursors are deposited (Johnson, 2015b). The process of follicle selection has been widely recognized as the rate-limiting step of the reproductive potential of birds (Johnson, 2015b). Hence, it is of great theoretic and practical importance to clarify the underlying mechanisms.

It is well accepted that follicle-stimulating hormone (FSH) plays a pivotal role in the control of avian follicular development and that associated differentiation following follicular selection depends on both FSH stimulation and intrafollicular factors (Kim and Johnson, 2018). The importance of various members of the transforming growth factor (TGF) β superfamily has been demonstrated. Among them, growth differentiation factor 9 (GDF9) promotes FSH-induced progesterone production and steroidogenic acute regulatory protein (STAR) expression in ovarian granulosa cells of chickens (Li et al., 2019). Bone morphogenetic protein (BMP)-4, BMP-6, and BMP-15 enhance FSH receptor (FSHR) mRNA expression in granulosa cells from prehierarchical follicles of hens (Ocon-Grove et al., 2012; Dongwon et al., 2013; Stephens and Johnson, 2016). Subsequently, enhanced FSH signaling facilitates the process of follicle selection (Johnson, 2015b). Since ovarian follicles are composed of a central oocyte and surrounding somatic cells (i.e., granulosa and theca cells), it is generally accepted that, in avian species, follicle selection is the result of coordinated interactions between oocyte and somatic cells in response to a variety of endocrine, paracrine, and/or autocrine factors (Johnson, 2015b; Li et al., 2019). In birds, several types of interactions have been suggested in the germinal-somatic regulatory loop, including paracrine signaling pathways, gap junctions, and other junctional contacts, which play a substantial role for the control of cell communication and oocyte maturation (Schuster et al., 2004; Stephens and Johnson, 2017; Li et al., 2019). In mammals, through intercellular contacts, granulosa cells, theca cells, and oocyte form a functional syncytium by which small molecules (up to 1 kDa in mass) are exchanged to meet the metabolic and regulatory demands of the oocyte (Coticchio et al., 2015). However, most previous studies in birds mainly focused on the role of granulosa cells during follicle selection, with a particular focus on ovarian cell differentiation and steroidogenesis capacity (Johnson, 2015b). This research has certain restrictions because these data present the consequence of follicular selection but not a proximal cause (Johnson and Woods, 2009). Furthermore, information on the actions of other properties of granulosa cells and their interactions with theca cells during the process of follicular selection as well as the underlying mechanisms remains limited. During the last 5 years, high-throughput sequencing technology has been employed to investigate the epigenetic and genetic mechanisms that control avian follicle development (Li et al., 2013; Xu et al., 2013; Wang et al., 2017). However, most of these studies were conducted on either the whole ovary (Li et al., 2013; Xu et al., 2013) or intact follicles (Liu et al., 2015; Wang et al., 2017) and were mainly aimed to explain the differences in egg-producing performance among different breeds of poultry or to identify the mechanisms that initiate egg production and broodiness (Xu et al., 2013). So far, neither the mRNA nor miRNA transcriptome profiling has been reported in either avian granulosa or theca layers. Therefore, a comprehensive understanding of the mRNA–miRNA interaction network in both granulosa and theca layers during follicle selection is required.

The present study investigated mRNA and miRNA transcriptome profiling in both granulosa and theca layers from geese follicles prior to selection (4–6 and 8–10 mm in diameter prehierarchical follicles) and after selection (F5 hierarchical follicles) through high-throughput RNA sequencing (RNA-seq). With the utilization of the Short Time-series Expression Miner (STEM) method, a number of protein-coding genes and miRNAs with similar expression patterns during follicle selection were identified and visualized. This was followed by Kyoto Encyclopedia of Genes and Genomes (KEGG) enrichment analyses of both these differentially expressed (DE) mRNAs and predicated target genes of miRNAs. Then, co-expression networks were built using Cytoscape to explore the interactions among either the node mRNAs alone or the mRNA:miRNA pairs associated with adhesion and junctions as well as lipid metabolism during follicle selection. These results provide a better understanding of the mechanisms underlying the process of follicle selection and aid the ultimate goal toward improving the reproductive capacity of poultry.

## Materials and Methods

### Experimental Animals and Sample Collection

**Figure 1:**
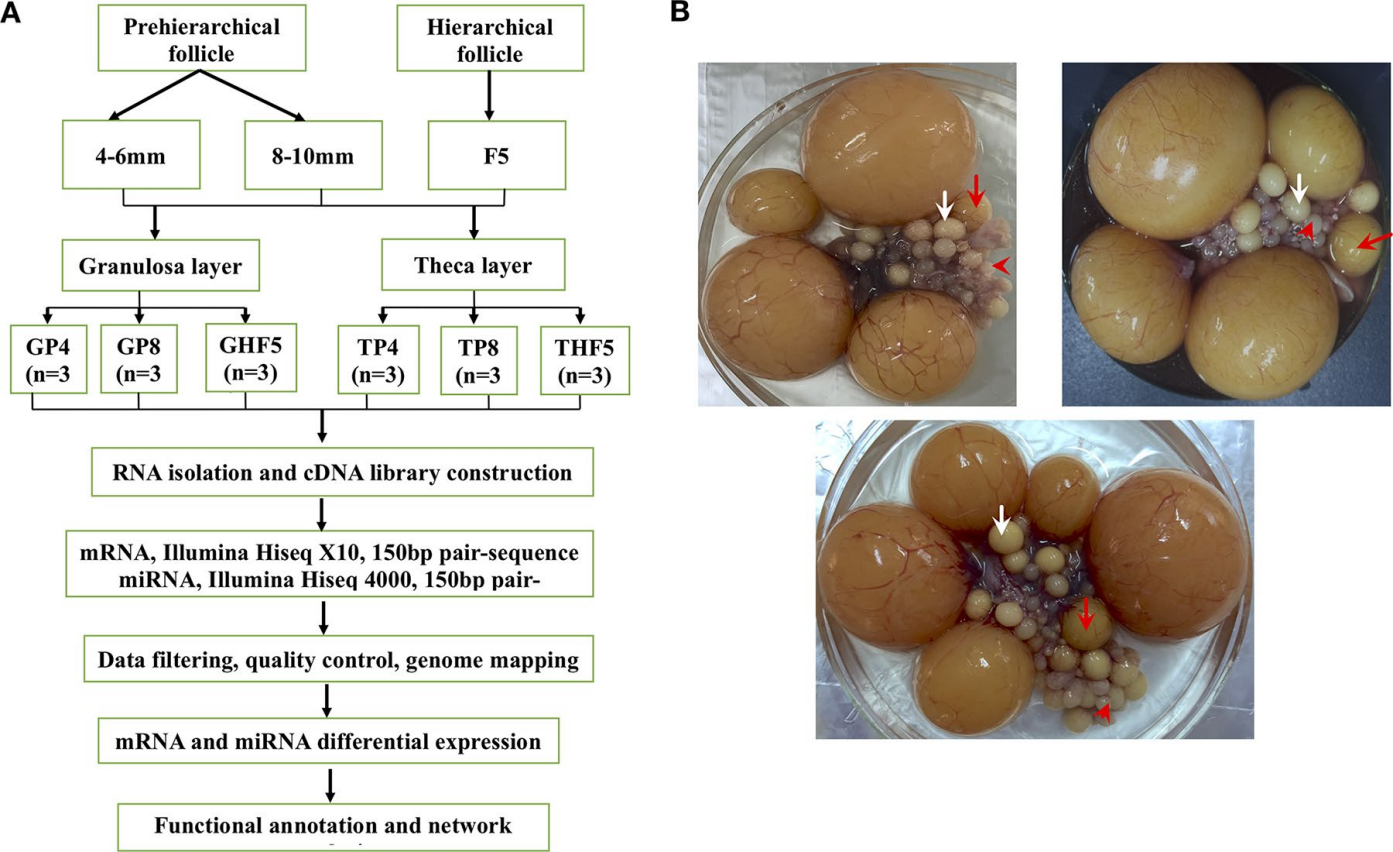
Experimental design **(A)** and sampling of geese ovarian follicles **(B)**. Cohorts of follicles include pre-hierarchical (follicles with diameters of 4–6 and 8–10 mm, marked by the red arrowheads and white arrows, respectively) and hierarchical follicles (F5 follicles, marked by red arrows) collected from three individual geese. Both granulosa and theca layers, separated from these follicles, were used for mRNA and small RNA sequencing, followed by a series of bioinformatics analyses. GP4, GP8, and GHF5 represent granulosa layers isolated from 4–6-mm, 8–10-mm, and F5 follicles, respectively. TP4, TP8, and THF5 represent theca layers isolated from the 4–6-mm, 8–10-mm, and F5 follicles, respectively.

The healthy maternal line of Tianfu meat geese (*Anser cygnoides*, 35–45 weeks) from the Experimental Farm for Waterfowl Breeding at Sichuan Agricultural University (Ya’an, Sichuan, China) was used in the present study. Detailed information on animals, laying cycles, and the classification criteria of ovarian follicles has been previously described (Hu et al., 2014). Granulosa and theca layers were separated from three cohorts of healthy follicles of three individual geese (including the 4–6-mm, 8–10-mm, and F5 follicles, with average follicle numbers per individual of 18 ± 3, 8 ± 2, and 1, respectively) according to previously described methods (Gilbert et al., 1977). All separated granulosa and theca layers (*n* = 18) were then subjected to both RNA isolation and sequencing, followed by a series of bioinformatic analyses (see detailed information in **Figure 1**). All experimental procedures that involved animal manipulation were approved by the Faculty Animal Care and Use Committee of Sichuan Agricultural University (Ya’an, Sichuan, China) under permit no. DKY-S20143204.

### RNA Isolation, Library Construction, and Sequencing

The total RNA was extracted from all collected samples using the miRNeasy Mini Kit (Qiagen, Hilden, Germany) following the manufacturer’s protocol. The RNA concentration was determined using the Qubit RNA Assay Kit (Life Technologies, Carlsbad, CA, USA). RNA integrity was analyzed on the Agilent 2100 Bioanalyzer System (Agilent Technologies, Palo Alto, CA, USA). Eighteen RNA samples were used for library construction in accordance with previously described procedures (Sun et al., 2015). The mRNA and small RNA libraries were sequenced by Shanghai OE Biotech Co., Ltd. (Shanghai, China) using Illumina HiSeq X10 and HiSeq 4000 (Illumina, San Diego, CA, USA), respectively.

### Quality Control, Mapping, and Annotation of RNA-Seq Reads

Raw data of RNA-seq were processed using the NGS QC Toolkit (Patel and Jain, 2012). Reads that contained poly-N and low-quality reads were removed to obtain clean reads. Then, these clean reads were mapped to the geese (*A. cygnoides*) reference genome (assembly Ans Cyg_PRJNA183603_ v1.0, https://www.ncbi.nlm.nih.gov/genome/31397?genome_assembly_id=229313) using Tophat 2 (Kim et al., 2013). The annotation of genes was obtained from AnsCyg_PRJNA183603_v1.0_genomic annotations files.

### PCA, Identification of DE Genes, and Clustering Analysis

Principal component analysis (PCA) was performed in the R package using ggplot2. The fragments per kilobase million (FPKM) and transcripts per million (TPM) values were used to quantify the mRNA and miRNA expression levels, respectively. A DE analysis between groups was conducted using DESeq (2012), which estimates both size factors and Nbinom test (Varet et al., 2016). *P* value < 0.05 and fold change (FC) > 2 or <0.5 were set as thresholds to identify significantly DE mRNAs, while a *P* value < 0.05 was used as threshold to identify DE miRNAs. Additionally, Venn diagrams were depicted for both DE mRNAs and miRNAs using the Limma package in R. Hierarchical clustering and STEM analyses were conducted to explore the expression profiles of both DE mRNAs and miRNAs during different stages of follicle development.

### Functional Enrichment and Interaction Network Analysis

KEGG enrichment analyses of DE genes were performed using the R package, based on the hypergeometric distribution. Co-expression networks were built to investigate interactions among either DE genes alone or among miRNA:mRNA pairs. Cytoscape (http://www.cytoscape.org/) was used to visualize potential mRNA–miRNA interaction networks.

### Identification, Renaming, and Target Gene Prediction of miRNAs

To categorize small RNAs and to identify novel miRNAs, the following trimming and identifying processes were performed: (1) reads with 5′ and 3′ sequencing adapters were eliminated with Cutadapt software; (2) small RNA libraries were further filtered to a minimum length of 18 nt and a maximum length of 41 nt; (3) clean tags were mapped to the goose genome (*A. cygnoides*) using MegaBLAST, and rRNA, tRNA, snoRNA, and snRNA were removed from small RNA sequences; (4) the remaining reads were used to identify known miRNAs *via* alignment against the miRNA sequence using miRBase v.21 database; and (5) unannotated small RNAs were analyzed by mirdeep2 to predict novel miRNAs. Since a multi-species miRNA database was adopted to identify miRNAs, to unify the name of miRNAs, merging and renaming of miRNA were conducted, as follows: (1) all predicted miRNAs were renamed according to *A. cygnoides*, such as “*acy*-novel-miR-1” or “*acy*-novel-miR-1*”; (2) all chicken miRNAs retained the original name; (3) the remaining known miRNAs of other species were merged and renamed according to their respective miRNA family, for example, “*acy*-miR-.” The highest expressed sequence was a representative sequence of the miRNA. Target genes of DE miRNAs were predicted with the software package Miranda.

### Quantitative Real-Time PCR

A total of 12 genes were selected for quantitative real-time polymerase chain reaction (qRT-PCR) to validate the obtained RNA-seq results. Reactions of qRT-PCR were performed with the SYBR PrimerScript™ real-time PCR kit (TaKaRa, Dalian, China) using the CFX96™ Real-Time system (Bio-Rad, Hercules, CA, USA). The specificity of each primer was verified with its standard curve (Svec et al., 2015). Each sample was run in triplicate, and the relative mRNA expression level was normalized using *GAPDH* and *β-actin* as housekeeping genes according to the 2^-ΔΔCt^ method (Livak and Schmittgen, 2001). The correlation between qRT-PCR and RNA-seq results was calculated with Microsoft Excel 2013. Both forward and reverse primers are listed in **Table S1**.

### Statistical Analysis

Analyses of the mRNA expression levels of related genes were subjected to analysis of variance (ANOVA), and the means were assessed for significant differences using the Student *t*-test. All results were expressed as means ± SD, and *P*-values below 0.05 were considered to indicate statistically significant differences. All statistical analyses were performed using SPSS software (version 21).

## Results

### Overview of the mRNA and miRNA Transcriptome of Geese Granulosa and Theca Layers

A total of approximately 1.07 Gb of mRNA raw reads were generated from 18 cDNA libraries through Illumina HiSeq X10 sequencing. After stringent filtering, an average of 57.8 million clean reads were generated per sample, and an average 70.06% of these clean reads were mapped to the *Anser cygnoides* 1.0 genome (**Table S2**). Under a cutoff of FPKM > 0.5, expression of 13,282 annotated genes (covering 63.90% of 20,787 gene set) was identified in at least one individual. The length distribution of mRNAs is illustrated in **Figure S1A**. After quality filtering of 258 million raw reads, 228 million clean miRNA reads from 18 samples remained for subsequent analyses. Of these reads, 83.86% had a length of 18–26 nt, with an average size of 22.84 nt (**Figure S1B**), and an average of 89.71% of the reads were mapped to the goose genome (*A. cygnoides* 1.0) (**Table S3**). The majority of small RNAs were known miRNA (approximately 66.55%), followed by unannotated RNA (approximately 20.51%) (**Figure S1C** and **Table S4**).

To ensure accurate comparisons between follicles from prehierarchical (4–6 and 8–10 mm) and hierarchical (F5), expression levels of *FSHR*, *CYP11A1*, *STAR*, *3β-HSD*, and *AMH* in the granulosa layer were detected by qRT-PCR. Their expression levels presented a significant up- or down-regulation in *F5* follicle compared with follicles with diameters of 4–6 and 8–10 mm and were similar to that observed using transcriptome sequencing (**Figure S2**). This suggests that the F5 follicle entered the hierarchical system on the basis of the fact that these genes are well-known markers for differentiation of granulosa cells after follicular selection (Johnson, 2012; Johnson, 2015b). Since *CYP17A1* and *CYP19A1* are almost exclusively expressed in avian theca cells (Kato et al., 1995; Gan et al., 2017), and *FSHR* is mainly present in avian granulosa cells (Woods and Johnson, 2005), expression of *CYP17A1*, *CYP19A1*, and *FSHR* was used to identify theca and granulosa cells. In the present study, we found that no more than 1% and 7% of cyp17a1 and cyp19a1 gene expression but more than 80% of FSHR were present in the granulosa layer (**Figure S2**). This indicates that granulosa and theca layers had been successfully separated in this study. In addition, another seven protein-coding genes, that is, a total of 12 protein-coding genes, were randomly selected for qRT-PCR to confirm the RNA-seq results. A high correlation in log2FC (*R*^2^ = 0.91) between RNA-seq and qRT-PCR was observed (**Figure S3**). Thus, these results confirm the high credibility of RNA-seq data in the present study.

### Larger Transcriptome Diversification in the Granulosa Layer During Follicle Selection

**Figure 2:**
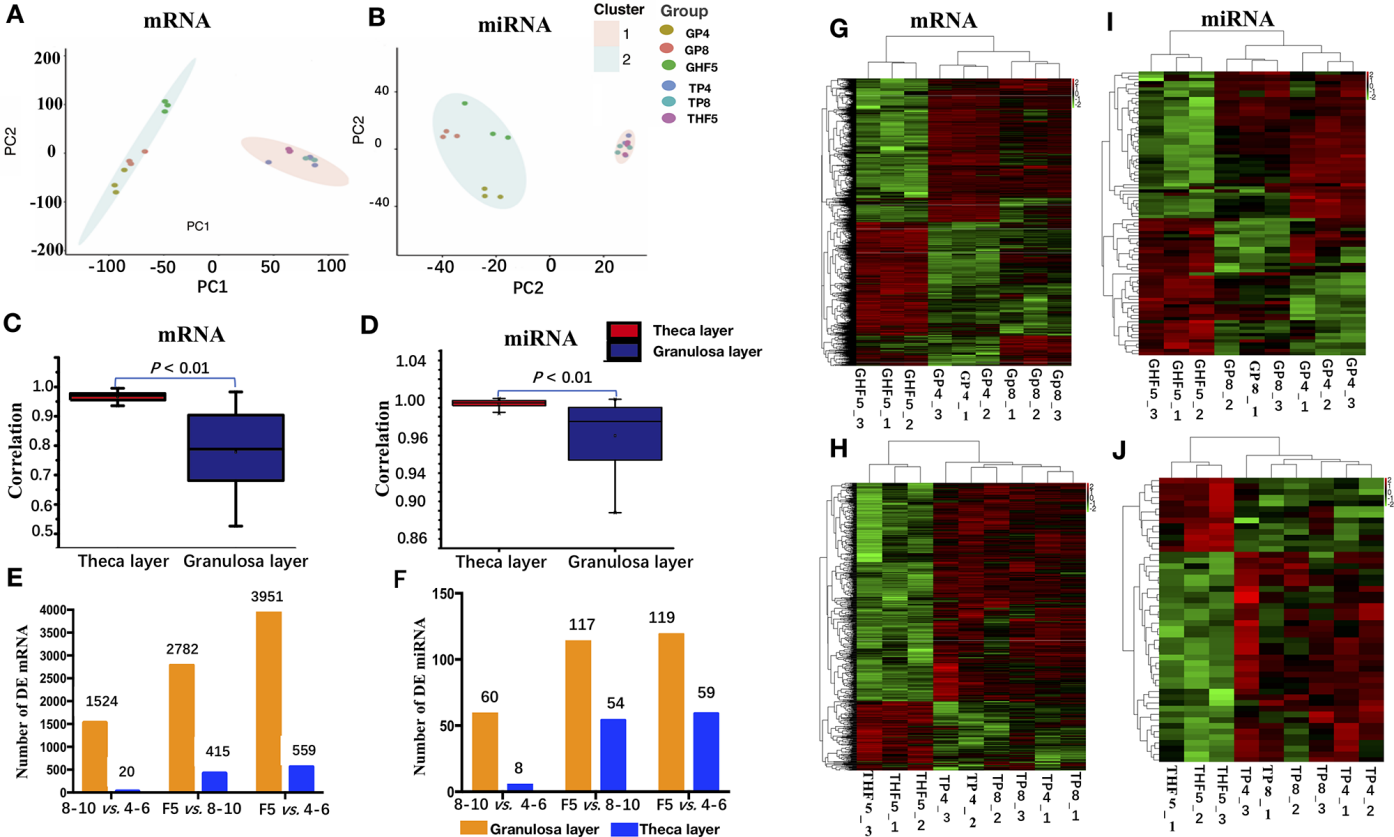
mRNA and miRNA transcriptome sequencing and identification of differentially expressed (DE) mRNAs and miRNAs in either granulosa or theca layers. **(A**, **B)**: Principal component analysis of the mRNA and miRNA transcriptomes among 18 libraries. **(C**, **D)** Pearson correlation analysis of the mRNA and miRNA transcriptome among nine granulosa or theca layers. **(E**, **F)** Number of DE mRNAs and miRNAs in either granulosa or theca layers. **(G**–**J)** Heatmap analysis of DE mRNAs **(G, H)** and miRNAs **(I, J)** in granulosa or theca layers. 4–6: follicles with 4–6 mm in diameter; 8–10: follicles with 8–10 mm in diameter; F5: hierarchical F5 follicle. GP4_1, GP4_2, and GP4_3 represent three replicates of GP4, and similar definitions have been used for GP8, GHF5, TP4, TP8, and THF5.

PCA results showed the two main characteristics (**Figures 2A, B**): (1) the three repeating samples were tightly clustered regardless of whether these were mRNAs or miRNAs, especially in the granulosa layer, indicating high experimental confidence in the obtained results; (2) both the granulosa layer and theca layer were distinctly separated by the first eigenvector for both mRNAs and miRNAs, showing varying degrees of divergence in both tissues. To further explore the transcriptome difference of both the mRNA and miRNA transcriptomes between granulosa and theca layers, their Pearson’s correlation coefficients between granulosa and theca layers were calculated (**Figures 2C, D**). The results showed that compared with the theca layer, the granulosa layer showed a wider range of Pearson’s correlation coefficient for both mRNAs (0.52 to 0.98) and miRNAs (0.87 to 0.99). This indicates that the granulosa layer presented smaller transcriptome consistency.

DE mRNAs and miRNAs were further screened during different stages of follicle development. A total of 2,782 DE mRNAs and 117 DE miRNAs were identified in the granulosa layer during follicle selection (i.e., F5 vs. 8–10-mm group), which is approximately twice the number of the DE genes (1,524 mRNAs and 60 miRNAs) that had been identified prior to follicle selection (i.e., 8–10-mm vs. 4–6-mm group). Similarly, for the theca layer, the number of DE genes in the F5 vs. 8–10-mm group (415 mRNAs and 54 miRNAs) exceeded that of DE genes in 8–10- vs. 4–6-mm group (20 mRNAs and 8 miRNAs) (**Figures 2E, F**). Clearly, more DE genes (mRNAs or miRNAs) were found in the granulosa layer than in the theca layer by observing the number of DE genes at any stage. Furthermore, a similar result was acquired by observing the number of uniquely up-regulated or down-regulated DE mRNAs and miRNAs during follicle selection using a Venn diagram (**Figure S4**). In addition, a hierarchical analysis indicated that the DE genes (both in the granulosa or theca layer) were firstly clustered between the follicles with 8–10 mm in diameter and those with 4–6 mm in diameter, followed by F5 follicles (**Figures 2G–J**). In conclusion, these results demonstrate that a higher transcriptome diversification is presented during follicle selection than prior to follicle selection, especially in the granulosa layer.

Based on log2FC, which ranged from -7.92 to 11.24, the top 20 up- and down-regulated mRNAs for the granulosa layer between F5 and 8–10 mm in diameter follicles were screened (**Table S5**). Among these, up-regulated genes included apolipoprotein B (*APOB*), fatty acid binding protein, liver and adipocyte (*FABP1* and LOC106030656, respectively), ATP-binding cassette 5 (*ABCB5*), and perilipin 1 (*PLIN1*), all of which were reported to be involved in lipid regulation (Beilstein et al., 2016). Furthermore, *STAR*, G protein-coupled receptor (*GPR68*), and progesterone receptor (*PGR*), which have been widely accepted as markers of steroidogenesis capability within granulosa cells after follicular selection (Walsh et al., 2012; Bishop et al., 2016), were also profoundly up-regulated in the granulosa layer. Among the top 20 down-regulated genes, collagens including type XXII alpha 1 (*COL22A1*) and alpha-1(I) chain-like (*LOC106046014*) were used as the basic composition of the extracellular matrix (ECM) (Woodruff and Shea, 2007). In contrast, log2FC of the top 20 up- and down-regulated genes from the theca layer in the F5 vs. 8–10-mm group only varied from -4.70 to 3.28 (**Table S6**). Among these genes, up-regulated genes of sushi-repeat protein X-linked 2 (*SRPX2*), neuron-derived neurotrophic factor (*NDNF*), and thrombospondin 1 (*THBS1*) were reportedly involved in endothelial cell migration and angiogenesis (Marijana et al., 2009; Koji et al., 2014; De Palma et al., 2017).

The miRNAs varied from -4.97 to 3.62 on the basis of log2FC, and 29 up-regulated and 33 down-regulated miRNAs from the granulosa layer in the F5 vs. 8–10-mm group were identified. Among the up-regulated miRNAs, *gga*-miR-29b-1-5p increased about 12-fold; *gga*-let-7c-3p, *gga*-miR-199-5p, and *acy-*miR-2954 increased >4.55-fold; and *acy-*miR-340 and *acy*-miR-378 decreased about fivefold and threefold in F5 follicles compared with 8–10-mm follicles, respectively (**Table S7**). In addition, nine up-regulated and 25 down-regulated DE miRNAs from the theca layer were identified between follicles F5 and 8–10 mm in diameter on the basis of the log2FC ranging from -2.15 to 1.36 (**Table S8**).

### Involvement of Junctional Adhesions and Lipid Metabolism During Follicle Selection

**Figure 3:**
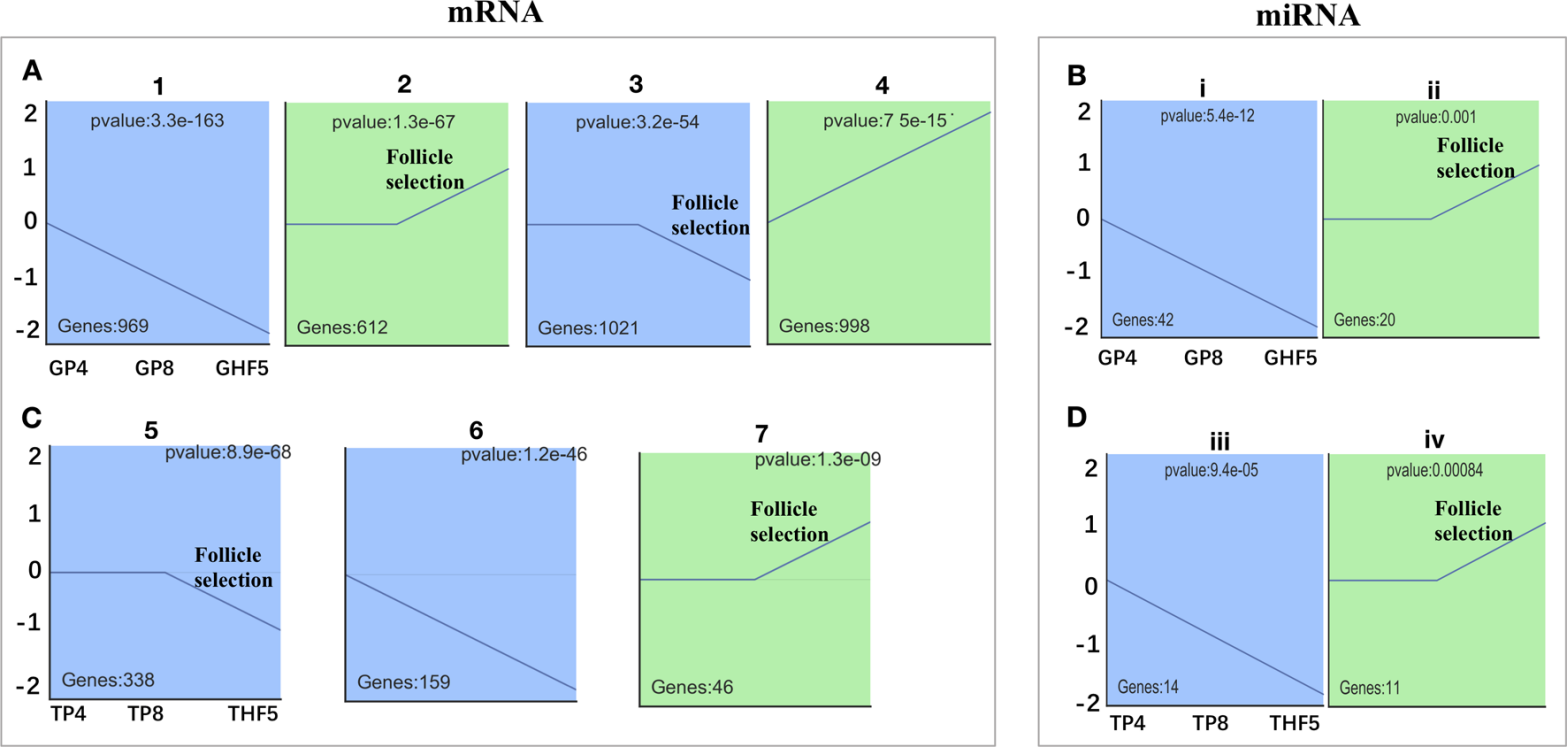
Screening of significantly differentially expressed (DE) mRNAs and miRNAs among either in granulosa or theca layers of geese follicles at different developmental stages. Time-series cluster analysis for the expression profiling of DE mRNAs and miRNAs in the granulosa layer **(A**, **B)** and theca layer **(C**, **D)**. Note that the transition period for follicles from 8–10 mm in diameter to F5 was used for follicle selection. Profiles 2, 3, 5, 7, ii, and iv indicated those DE mRNAs or miRNAs with unchanged expression before follicle selection, following significant fluctuation during follicle selection. Profiles 1, 4, 6, i, and iii represent DE mRNAs or miRNAs with significantly changed expression throughout follicle development.

**Figure 4:**
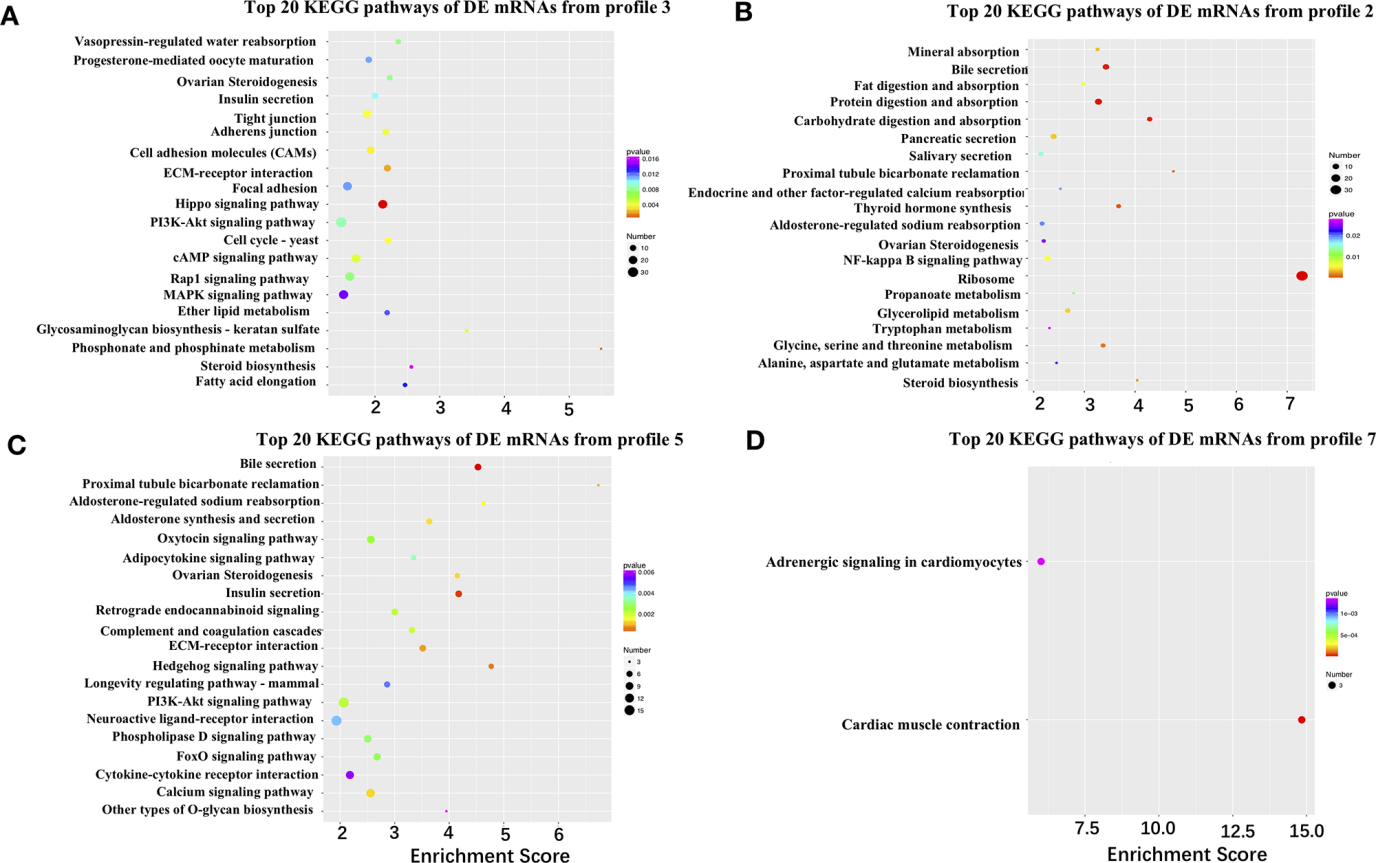
Kyoto Encyclopedia of Genes and Genomes (KEGG) pathways of differentially expressed (DE) mRNAs from different time-series modules in either granulosa or theca layers. **(A**, **B)** Top 20 KEGG pathways of DE mRNAs from profile 3 **(A)** and profile 2 **(B)** in the granulosa layer. Five pathways of tight junction, adherens junction, cell adhesion molecules, ECM–receptor interaction, and focal adhesion were identified in profile 3 **(A)**. The two pathways of fat digestion and absorption and glycerolipid metabolism were contained in profile 2 **(B)**. **(C**, **D)** Top 20 KEGG pathways of DE genes of profile 5 **(C)** and profile 7 **(D)** in the theca layer.

To further narrow down the list of genes that might be involved in follicle selection, a STEM analysis was performed with the DE mRNAs screened by RNA-seq. As shown in **Figure 3**, seven significant profiles for mRNAs (profiles 1–4 in the granulosa layer and profiles 5–7 in the theca layer) and four significant profiles for miRNAs (profile i and ii in the granulosa layer and profile iii and iv in the theca layer) were identified according to the *P*-values. Among mRNAs, four significant profiles clustered during follicle selection; thus, a functional analysis mainly focused on the expression patterns for profiles 2 and 3 of the granulosa layer (**Figure 3A**), as well as profiles 5 and 7 of the theca layer (**Figure 3C**), whose expression levels remained unchanged prior to follicle selection and then either increased or decreased during follicle selection (**Figure S5**). For profile 3, five of the top 20 significant KEGG pathways were involved with biological processes of junctional adhesions such as “tight junction” (TJ), “adherens junction,” “cell adhesion molecules” (CAMs), “ECM–receptor interaction,” and “focal adhesion” (**Figure 4A**). For profile 2, two of the top 20 pathways were related to the processes of “fat digestion and absorption” and “glycerolipid metabolism” (**Figure 4B**). Moreover, pathways of “ovarian steroidogenesis” and “steroid biosynthesis” were also significantly enriched during the process of follicular selection (**Figures 4A, B**). Profile ii of miRNAs showed the opposite trend with profile 3 of mRNAs in the granulosa layer and so did profile iv of miRNAs and profile 5 of mRNAs in the theca layer during follicle selection (see **Figures 3B, D**). Functional enrichment analyses of predicted target genes of miRNAs from profiles ii and iv were performed. Interestingly, “ECM–receptor interaction” and “focal adhesion” were identified during follicle selection (**Figure S6**). Combing these results suggests that pathways of junctional adhesion, lipid metabolism, and ovarian steroidogenesis in the granulosa layer play an important role for the regulation of follicular selection. For the theca layer, functional enrichment of down-regulated DE genes from profile 5 indicated that pathways of “neuroactive ligand-receptor interaction,” “complement and coagulation cascades,” and “cytokine-cytokine receptor interaction” were mainly included in the top 20 pathways (**Figure 4C**). However, up-regulated DE genes from profile 7 were enriched in two significant pathways of “adrenergic signaling in cardiomyocytes” and “cardiac muscle contraction” during follicle selection (**Figure 4D**).

### Network Analysis of DE mRNAs and miRNAs Related to Adhesion and Lipid Metabolism During Follicle Selection

**Figure 5:**
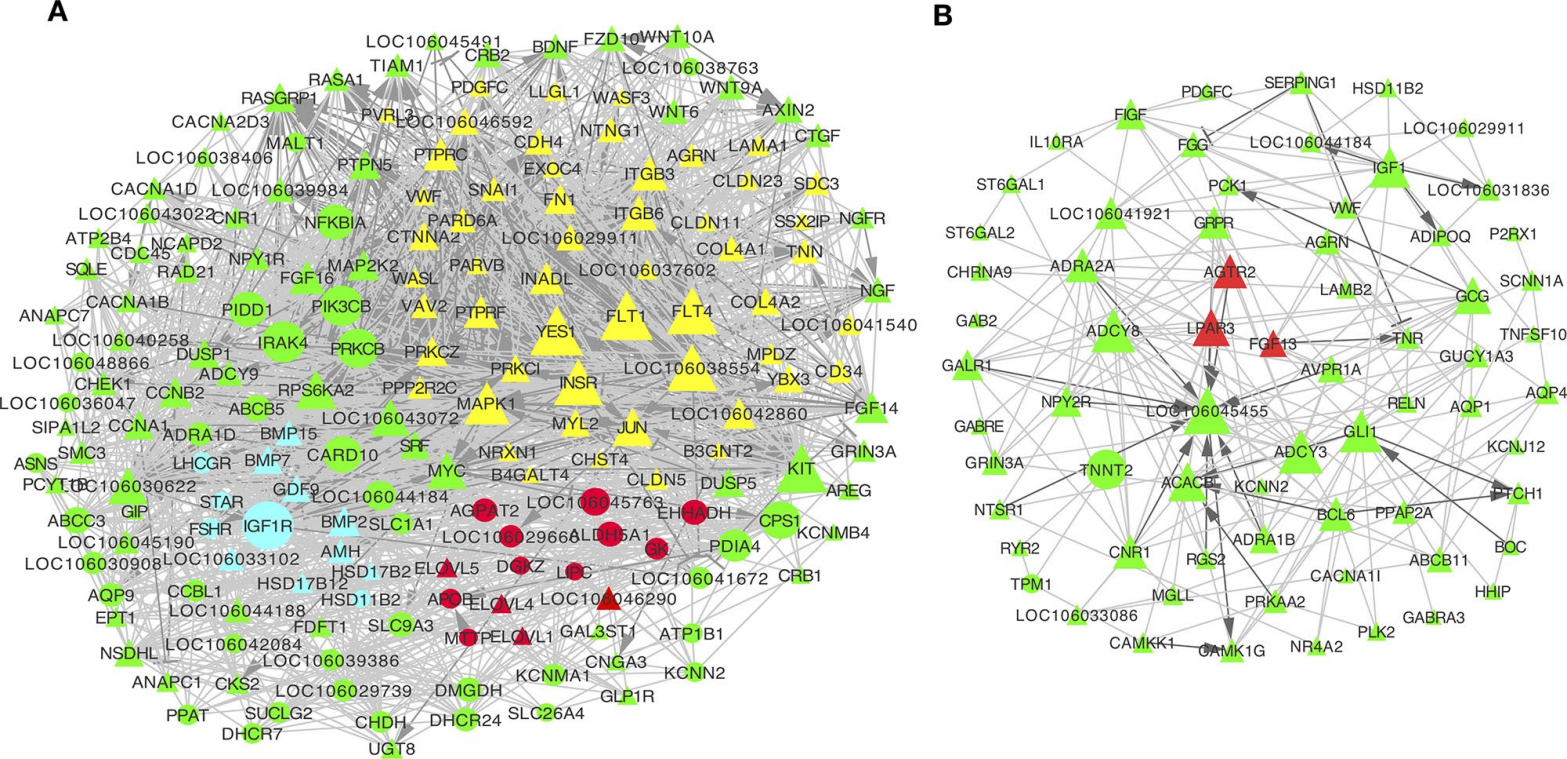
Network analysis of differentially expressed (DE) mRNAs in granulosa and theca layers. **(A**, **B)** Co-expression network of DE mRNAs from top 20 Kyoto Encyclopedia of Genes and Genomes (KEGG) pathways of profiles 2 and 3 in the granulosa layer **(A)**, as well as of profiles 5 and 7 in the theca layer **(B)**. This interaction network includes 53 junctional adhesion-related genes (marked in yellow), 15 lipid metabolism-related genes (marked in red), and 13 genes that are involved in ovarian steroidogenesis (marked in blue) **(A)**. Genes marked with triangles were down-regulated, and those marked with circles were up-regulated during follicle selection. Solid lines indicated direct interactions. Dark grey genes had interactions of activation or inhibition, while light grey lines had interactions without activation or inhibition. Activation →; Inhibition.

To further explore the interactions among DE genes, a novel interaction network was constructed using Cytoscape software. Genes were selected that were enriched in the top 20 KEGG pathways from profiles 2 and 3 for the granulosa layer, as well as from profiles 5 and 7 for the theca layer (see **Figure 4**). While significant changes were found in their expression levels during follicle selection, levels remained unchanged before follicle selection. In the granulosa layer, this network showed that interactions mainly occurred among either 53 junctional adhesion-related or 15 lipid regulation-related genes (**Figure 5A**). The 53 genes in the network related to junctional adhesion mainly included fibronectin 1 (*FN1*), collagen type IV alpha 1 (*COL4A1*), collagen type IV alpha 2 (COL4A2), integrin beta 3 (*ITGB3*), laminin alpha 1 (*LAMA1*), agrin (*AGRN*), and claudins (*CLDN*s) (*CLDN5*, *CLDN11*, and *CLDN23*), whose expression levels presented a concurrent down-regulation during follicle selection. In addition, 14 hub genes involved in lipid regulation, such as *APOB*, microsomal triglyceride transfer protein (*MTTP*), 1-acylglycerol-3-phosphate O-acyltransferase 2 (*AGPAT2*), elongation of very long chain fatty acids (*ELOVL1*, *ELOVL4*, and *ELOVL5*), aldehyde dehydrogenase 5 family member A1 (*ALDH5A1*), and glycerol kinase (*GK*) were present in this network. Furthermore, up-regulated genes in the network such as insulin-like growth factor 1 receptor (*IGF1R*), *FSHR*, *STAR*, and luteinizing hormone/choriogonadotropin receptor (*LHCGR*). The down-regulated genes that belong to the TGF-β superfamily such as anti-Mullerian hormone (*AMH*), BMPS (*BMP-2*, *BMP-7*, and *BMP-15*), and *GDF-9* (**Figure 5A**) were proved to exert an essential role in the regulation of differentiation and steroidogenesis within granulosa cells following follicular selection (Baumgarten et al., 2014; Johnson, 2015b; Chang et al., 2019; Li et al., 2019). Therefore, *via* further screening, DE mRNAs involved in adhesion, lipid regulation, and steroidogenesis based on the pool of differential expressed protein-coding genes were identified and visualized by heatmap clustering (**Figure S7**). This result indicated that an apparent dynamic change in expression level was present in the transition stage from 8–10 mm in diameter to F5 follicle. However, this retained an almost constant level in transitional follicles from 4–6 mm to 8–10 mm in diameter, suggesting that these genes participate in modulating follicular selection.

Furthermore, the interactions among those genes enriched in the top 20 KEGG pathways from profile 5 and 7 of the theca layer were also predicted. Most of them were down-regulated during follicle selection. Of these, three hub genes involving angiotensin II receptor, type 2 (*AGTR2*), fibroblast growth factor 13 (*FGF13*), and lysophosphatidic acid (LPA) receptor 3 (*LPAR3*) (**Figure 5B**, marked by red color) have been reported to participate in cell migration and angiogenesis in bladder cancer or lactotroph tumors (Pei et al., 2017; Wierinckx et al., 2018; Chen et al., 2019).

**Figure 6:**
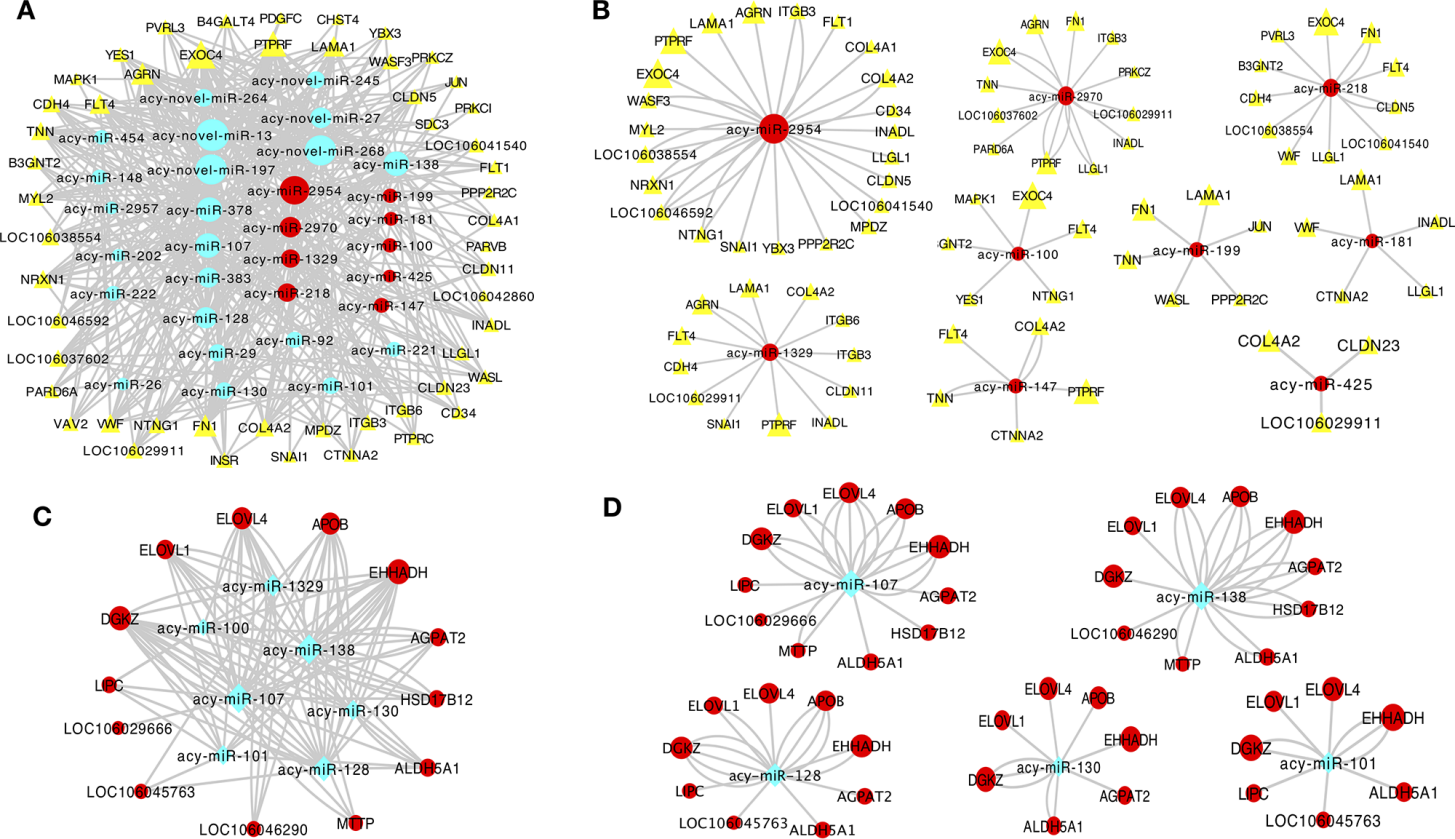
Co-expression network analysis of differentially expressed (DE) miRNAs and mRNAs in relation to junctional adhesion **(A**, **B)** and lipid metabolism **(C**, **D)** in the granulosa layer. The interaction networks among 31 miRNAs and 53 mRNAs associated with junctional adhesion **(A)**, and among these miRNAs, *acy*-miR-2954, *acy*-miR-2970, *acy-*miR-1329, *acy*-miR-218, *acy*-miR-199, *acy*-miR-100, *acy*-miR-181, *acy*-miR-147, and *acy*-miR-425 contained 23, 12, 12,11, 6, 6, 5, 5, and 3 protein-coding genes related to junctional adhesion, respectively **(B)**. Seven miRNAs and 13 mRNAs were related to lipid metabolisml **(C)**, and among these miRNAs,: *acy*-miR-107, *acy*-miR-138, *acy*-miR-128, *acy*-miR-130, and *acy*-miR-101 interacted with 11, 10, 9, 7, and 7 protein-coding genes implication in lipid regulation, respectively **(D)**.

Co-expression networks were established to investigate the association between the expression of DE mRNAs and miRNAs in the granulosa layer related with junctional adhesion and lipid metabolism (**Figure 6**) during follicle selection. Interaction networks between DE miRNAs and mRNAs were constructed using Cytoscape software. Co-expression networks containing 31 miRNAs and 53 protein-coding genes related with junctional adhesion were identified in the granulosa layer during follicle selection, among which 25 were known miRNAs and six were novel miRNAs (**Figure 6A**). Of all known miRNAs, expression of nine miRNAs (*acy*-miR-2954, *acy*-miR-218, *acy*-miR-2970, *acy-*miR-100, *acy*-miR-1329, *acy*-miR-199, *acy*-miR-425, *acy*-miR-147, and *acy*-miR-181) increased by about twofold during follicle selection (**Table S7**, marked by yellow colors). These presented an inverse relationship with adhesion-related mRNAs; thus, nine miRNA–mRNA interaction pairs related with junctional adhesion were constructed (**Figure 6B**). In addition, a network with seven known miRNAs and 13 protein-coding genes related with lipid metabolism were also identified (**Figure 6C**). Of these, the expressions of *acy*-miR-107, *acy*-miR-138, *acy*-miR-128, *acy*-miR-130, and *acy*-miR-101 (**Table S7**, marked by blue colors) changed inversely with most lipid regulation-related protein-coding genes wherein the five miRNA–mRNA interaction pairs were built (**Figure 6D**).

## Discussion

The present study is the first to systematically investigate the expression profiling of mRNA and miRNA transcriptomes in both granulosa and theca layers from follicles before and after selection in birds. Particular attention was focused on the identification of central candidate genes, signaling pathways, and hub genes of either the mRNA-mRNA or miRNA–mRNA interaction network for follicle selection regulation. PCA and qRT-PCR analyses demonstrated the high credibility of transcriptome sequencing achieved in this study. Additionally, PCA also indicated that the granulosa or theca layers were clustered separately. Furthermore, far more DE genes were identified in the granulosa layer than in the theca layer, which was similar to the results for horses and bovines, where more DE genes were observed in the granulosa layer than in the theca layer *via* transcriptome profiling (Donadeu et al., 2014; Hatzirodos et al., 2015). This study identified approximately twice the number of DE mRNAs and miRNAs during follicle selection compared with their number prior to follicle selection. Furthermore, hierarchical clustering showed higher transcriptome divergence in both granulosa and theca layers during follicle selection than before follicular selection. Similarly, increased transcriptome diversity was observed in granulosa cells from dominant follicles compared with those from subordinate follicles in bovines and horses (Donadeu et al., 2014; Gebremedhn et al., 2015). Hence, in combination with previously published results, the obtained results indicate that compared with the theca layer, the granulosa layer may play a more important role in the regulation of follicle development, especially during follicle selection.

The STEM analysis of all DE genes further screened for genes with uniquely changed expression during follicle selection. A downstream functional analysis of genes that originated from the granulosa layer indicated that they were involved in biological processes of junctional adhesion including “ECM–receptor interaction,” “cell adhesion molecules”, “tight junction”, “adherens junction,” and “focal adhesion.” Furthermore, pathways involving “ECM–receptor interaction” and “focal adhesion” were also enriched in the predicted target genes of miRNAs that presented changes contrasted with those of mRNAs in the granulosa layer during follicle selection. Construction of a co-expression network identified 53 hub genes related with the five pathways of junctional adhesions (e.g., *FN1*, *COL4A*s, *ITGB3*, *LAMA1*, and *CLDN*s). Interestingly, they all were consequently down-regulated. In short, the obtained data suggest that protein-coding genes associated with junctional adhesions play an important role in the regulation of follicular selection. Consistently, Donadeu *et al.* reported in a transcriptome profiling study that a large number of down-regulated DE genes in granulosa cells from horse ovarian follicles were enriched in the ECM during follicle dominance (Donadeu et al., 2014). Ożegowska *et al.* also reported genes enriched in cell adhesion as new potential markers by analyzing the transcriptome of porcine granulosa cells (Ożegowska et al., 2019). The ECM and ECM protein form a complex scaffold of protein that provides the architectural support for cells. ECM is mainly composed of *FN*, laminins (*LAMA*), and collagens such as *COL4A*s and perlecan (Rodgers et al., 2003). *FN*, laminin, and collagens (*COL4A1* and *COL4A4*) have been suggested to increase cellular attachment, expression of *FSHR*, and progesterone production in cultured porcine granulosa cells (Sites et al., 1996). Furthermore, these genes also regulate the survival, proliferation, and steroidogenesis of granulosa cells during follicle development (Woodruff and Shea, 2007). Cells are able to adhere to the ECM *via* different types of receptors such as integrins (*ITGB*s). For instance, laminin-α6β1 and integrin interaction enhances both the survival and proliferation to modulate steroidogenesis of ovine granulosa cells (Le et al., 2002). The *FN*-integrin pathway initiates the induction of granulosa cell luteinization and cumulus expansion during the ovulation process in mice (Kitasaka et al., 2018). TJs, including occludin (*OCLN*) and *CLDN*s, control the transport of water, ions, and macromolecules across cell layers (Suzuki, 2013). Thus, a model is proposed suggesting that hormonal down-regulation of TJ proteins during follicle development could decrease the selective permeability of molecules between follicular cells, thus resulting in an increase in the volume of follicular fluid into the oocyte, which may directly impact the functions of follicle maturation (Zhang et al., 2018). In hens, TJ protein (occludin) presents a stage-dependent decreasing expression pattern with follicle growth, thus suggesting that occludin forms a diffusion barrier between granulosa cells and oocytes. Immediately after follicle selection, the rapid and high capacity transport of yolk components into the oocyte can be found through a paracellular pathway (Stephens and Johnson, 2017). More intriguingly, the abundance and characteristics of gap junctions and adherens can vary under different phases and conditions during follicle development, especially during follicle selection. For example, human gap junctions and adherens are particularly numerous and form deep invaginations at the points of contact with the oolemma at the pre-antral stage; however, during the following antral stages, their number and complexity of contact at the oocyte surface decrease (Chang et al., 2016). Thus, interaction and communication between granulosa cells and oocytes depend on the composition of ECM with which it is implemented. These can differ over time in response to challenges of the developing follicle (Coticchio et al., 2015). Consistent with the existing results, the present data also indicated that genes in relation to ECM and junctional adherens in granulosa layer were also decreased in response to follicle selection, suggesting that down-regulation of these genes may contribute to a reduced selective permeability of molecules between somatic-oocyte and enable several molecules such as vitellogenin derived from hepar-synthesized to diffuse though the granulosa cells and act on the oocyte *via* paracrine regulation, thus ultimately promoting rapid oocyte growth as soon as the follicle is selected.

Moreover, the pathways that involve fat digestion and absorption, as well as glycerolipid metabolism, were found to be more active in hierarchical follicles, and genes such as *APOB*, *FABP1*, *FLIN1*, *ABCB5*, *AGPAT2*, *ELOVL*s, *ALDH5A1*, and *GK* associated with fatty acid synthase, lipid transportation, fatty acid elongation, lipid droplet formation, and triglyceride synthesis (Beilstein et al., 2016) were substantially up-regulated during follicle selection. In bovine granulosa cells, it has been demonstrated that lipid metabolism could affect both cell proliferation and progesterone synthesis (Elis et al., 2015). Moreover, several genes that are involved in fatty acid synthesis, lipoproteins degradation, *de novo* lipogenesis (DNL), and lipid accumulation were identified in bovine granulosa and theca cells using transcriptomic sequencing (Bertevello et al., 2018). In chickens or ducks, expression of acetyl-CoA dehydrogenase long chain (*ACADL*, which is involved in beta-oxidation of fatty acids), lecithin-cholesterol acyltransferase (*LCAT*, which is related to lipid catalysis), and apolipoprotein A-1 (*APOA1*, which is associated with lipid transport) were found to be elevated in birds with higher egg production rate than those with lower egg production rate (Yang et al., 2008; Wu et al., 2016). Furthermore, several genes that involve DNL, such as *FAS*, *ACC*, and *PPARγ* (in the present study, *PPARγ* was also expressed in granulosa layer of geese follicles and was significantly up-regulated during follicle selection according to qRT-PCR and RNA-seq results, see **Figure S2**), are expressed in geese ovarian follicles at different stages of development. This demonstrates the involvement of DNL during avian follicle development, especially during follicle selection (Wen et al., 2018). These findings further support the suggestion for mammals, where lipid metabolism is assumed to be essential for follicle development and oocyte energy supply (Dunning et al., 2014; Prates et al., 2014). Therefore, this study showed that in geese, those genes related to lipid regulation may play a significant role in the regulation of lipid deposition of yolk during follicle selection. Since the transfer of lipid-rich egg yolk into ovarian follicles is directly related to the egg production rate in birds (Barber et al., 1991; Stephens and Johnson, 2017), genes related to lipid regulation granulosa cells are proposed to be associated with low annual egg production in geese compared with chickens and ducks where egg formation required less lipid deposition.

In addition to the granulosa layer, many DE genes in the theca layer were also found during follicular selection. All of the up-regulated genes were enriched in the biological process of “cardiac muscle contraction” and “adrenergic signaling in cardiomyocytes.” It has been reported that typical smooth muscle cells are present in sow theca externa, which could be responsible for the contraction of ovarian theca cells, eventually assisting in ovulation or causing follicular collapse (O’Shea, 1970; Richards et al., 2018). Then, further screening DE genes in theca layer identified hub genes such as *SRPX2*, *NDNF*, *THBS1*, *FGF13*, *AGTR2*, and *LPAR* during follicular selection. *SRPX2* reportedly acts as a regulator of angiogenesis (Marijana et al., 2009); *NDNF* functions as a modulator that enhances endothelial cell function and revascularization processes (Koji et al., 2014); *THBS1* mediates IGF-I-induced steroidogenesis and proliferation in cultured porcine granulosa cells (Grado-Ahuir et al., 2009), inhibits angiogenesis, and promotes follicular atresia in rats (Garside et al., 2010); the *FGF*/*FGF* receptor contributes to tumor vascularization and adult angiogenesis (Presta et al., 2005); and lysophosphatidic acid plays a role in angiogenesis and enhances the angiogenic capability by the LPA1/3 receptor (Chen et al., 2019). Follicular steroidogenesis and survivability primarily depend on ovarian blood supply, and in mammals, vascularization has been reported to be an essential step in the modulation of follicle and corpus luteum development and function (Young and McNeilly, 2010; Berisha et al., 2016; Mishra et al., 2016). In summary, based on existing research results, changes in expression level of *SRPX2*, *NDNF*, *THBS1*, *FGF13*, *AGTR2*, and *LPAR3* in the theca layer during follicular selection may be important for a concurrent regulation in vasculature following follicular selection. Thus, this ultimately results in selected follicles consuming large amounts of lipid- and xanthophyll-rich yolk *via* an extensive vasculature (Johnson, 2015b).

In the current research, *PGR*, *IGF1R*, *FSHR*, and *STAR* were also found to be substantially up-regulated in the granulosa layer during follicular selection. It is worth noting that these genes are well-known markers for differentiation of granulosa cells and are closely involved with steroidogenesis after follicular selection (Baumgarten et al., 2014; Johnson, 2015b). For instance, IGF1R signaling is necessary for the FSH-induced activation of AKT and differentiation of human cumulus granulosa cells (Baumgarten et al., 2014). Additionally, the present data indicated that TGF-β superfamily members such as *AMH*, *BMP-2*, *BMP-7*, *BMP-15*, and *GDF-9* were down-regulated in granulosa layer after follicular selection. *AMH* was shown to regulate follicle selection by decreasing FSH sensitivity and inhibiting FSH-induced aromatase activity (Dewailly et al., 2016). *BMP-2* and *BMP-4* contribute toward maintaining granulosa cells of prehierarchical follicles in an undifferentiated state, and inhibiting FSH responsiveness in hen granulosa cells (Haugen and Johnson, 2010; Dongwon et al., 2013). *GDF-9* and *BMP-15* regulate growth, differentiation, and function of both granulosa and thecal cells during follicular development, inhibit FSH-stimulated progesterone secretion, and attenuate FSH-induced LH receptor formation in rat follicles (Vitt et al., 2000; Sanfins et al., 2018). However, *GDF-9* promotes FSH-induced progesterone production in chicken follicular granulosa cells (Li et al., 2019). In summary, in the present study, the reduced expression level of TGF-β superfamily members after follicle selection appears to exert an inhibiting effect imposed by the undifferentiated granulosa cells of prehierarchal follicles. This results in FSH-induced expression of downstream target genes such as *LHR*, *PGR*, and *STAR*, which ultimately promotes the conversion from undifferentiated prehierarchal to differentiated hierarchal follicles.

Since interactions between protein-coding genes and miRNAs are necessary to maintain normal ovarian activity (Maalouf et al., 2016), this study attempted to construct a co-expression network among mRNA:miRNA pairs, associated with junctional adhesion and lipid metabolism in the granulosa layer during follicle selection. A network analysis indicated that expression profiling of several node miRNAs (such as *acy*-miR-2954, *acy*-miR-2970, *acy*-miR-218, *acy*-miR-100, *acy*-miR-1329, *acy*-miR-199, *acy*-miR-425, *acy*-miR-181, and *acy*-miR-147) was closely related to junctional adhesion. Previous studies have shown that these miRNAs play a vital role in the regulation of cell migration and invasion by targeting adhesion-related genes (Yamamoto et al., 2013; He et al., 2016; Koshizuka et al., 2017). For instance, miR-218 can inhibit cancer cell metastasis and invasion by targeting *LAMB3* in cervical squamous cells (Yamamoto et al., 2013). miR-199 has been reported to regulate *FN* expression (Lee et al., 2009) and to inhibit cell migration and invasion in head and neck cancer by regulating *ITGA3* (Koshizuka et al., 2017). Moreover, miR-425 can inhibit hepatocellular carcinomas by targeting the cell–cell adhesion gene of *CTNNA3* (He et al., 2016). Furthermore, the present study showed that five miRNAs (*acy*-miR-107, *acy*-miR-138, *acy*-miR-130, *acy*-miR-128, and *acy*-miR-101) could be involved in regulating lipid metabolism. For instance, miR-107 is up-regulated in obese mice, and silencing of miR-107 leads to improved glucose homeostasis and insulin sensitivity (Trajkovski et al., 2011). miR-138 has been shown to negatively influence the adipogenic differentiation (Yang et al., 2011). miR-130 suppresses adipogenesis by inhibiting *PPARγ* expression (Lee et al., 2011). Thus, changes in the expression levels of the miRNAs related to either junctional adhesion (*acy*-miR-2954, *acy*-miR-2970, *acy*-miR-218, *acy*-miR-100, *acy*-miR-1329, *acy*-miR-199, *acy*-miR-425, *acy*-miR-181, and *acy*-miR-147) or lipid metabolism (*acy*-miR-107, *acy*-miR-138, *acy*-miR-130, *acy*-miR-128, and *acy*-miR-101) may exert an important regulatory function in the process of follicle selection. In addition, several novel miRNAs that interacted with adhesions- and lipid metabolism-related mRNAs during follicle selection were also identified in this study. Their physiological functions will be further investigated in the future.

## Conclusions

The present study examined the mRNA and miRNA transcriptome profiling in both granulosa and theca layers from geese ovarian follicles before and after selection. A large number of DE protein-coding genes and miRNAs associated with follicle selection were identified, especially in the granulosa layer. A functional enrichment analysis indicated that most of these DE genes that originated from granulosa layer were enriched in two groups of pathways: junctional adhesion and lipid metabolism. Additionally, a co-expression network was constructed to visualize interactions among protein-coding genes that are related to junctional adhesion and lipid metabolism, and an interaction network between relevant central mRNAs and miRNAs was also visualized. These data provide novel insights into the mechanisms underlying avian follicle selection. These newly identified candidate protein-coding genes and miRNAs associated with junctional adhesion and lipid metabolism, as well as the established miRNA–mRNA interaction network, lay a useful foundation for the improvement of the reproductive capacity of domestic poultry such as geese.

## Data Availability Statement

RNA- and miRNA-seq data used in the current study are available at the Sequence 557 Read Archive, BioProject PRJNA506334 (https://www.ncbi.nlm.nih.gov/bioproject/PRJNA506334).

## Ethics Statement

The animal study was reviewed and approved by Faculty Animal Care and Use Committee of Sichuan Agricultural University.

## Author Contributions

JW, SH, and QL conceived and designed this study. QL, YW, YD, and SY performed the experiments and analyzed the data. JH managed the experimental animals. QL and SH drafted the manuscript. JW and LL reviewed and improved this manuscript.

## Funding

This research was funded by the National Natural Science Foundation of China (No. 31672424 and 035Z2425), the China Agricultural Research System (No. CARS-42-4), the Open Fund of Farm Animal Genetic Resources Exploration and Innovation Key Laboratory of Sichuan Province (SNDK-KF-201802), the Project of National Science and Technology Plan for the Rural Development in China (No. 2015BAD03B06), and the Key Technology Support Program of Sichuan Province (No. 2016NYZ0044).

## Conflict of Interest

The authors declare that the research was conducted in the absence of any commercial or financial relationships that could be construed as a potential conflict of interest.

## Supplementary Materials

Figure S1 Basic information of RNA-seq and small RNA-seq. (A) Read length distribution of mRNAs; (B) read length distribution of miRNAs; (C) count distribution of total reads (left) and unique small RNAs (right). GP4_1, GP4_2, and GP4_3 represent three replicates of GP4, respectively, and similar definitions were used for GP8, GHF5, TP4, TP8, and THF5.

Figure S2 Confirmation of differentially expressed (DE) genes of RNA-seq by qRT-PCR. Mean ± SEM expression of FSHR, CYP11A1, STAR, 3β-HSD, AMH, and PPARγ were quantified in the granulosa layer by RNA-seq and qRT-PCR (A); CYP17A1, CYP19A1, and FSHR were identified in both the granulosa and theca layers by RNA-seq (B) isolated from 4-6-mm (n = 18 ± 3), 8-10-mm (8 ± 2), and F5 (n = 1) follicles. a and b represent significant differences among granulosa layers from three different stages based on the expression of these genes by RT-PCR (Student’s t-test, P < 0.05). ** indicates significant differences between granulosa and theca layer of three different stages (Student’s t-test, P < 0.01). SPSS software was used for statistical analysis.

Figure S3 Correlation between the results of RNA-seq and qRT-PCR.

Figure S4 DE mRNAs and miRNAs in the granulosa and theca layers. (A-C): The uniquely up- and down-regulated DE mRNAs between in the GHF5 vs GP8 group and GP8 vs GP4 group (A), and in the THF5 vs TP8 group and TP8 vs TP4 group (C). (B-D): The uniquely up- and down-regulated DE miRNAs between in the GHF5 vs GP8 group and GP8 vs GP4 group (A), and in the THF5 vs TP8 group and TP8 vs TP4 group.

Figure S5 Significant time-series modules of DE mRNAs in the granulosa and theca layers. Four significant time-series clusters were identified, including profiles 2 and 3 in the granulosa layer (A-B), as well as profiles 5 and 7 in the theca layer (C-D).

Figure S6 KEGG pathway enrichment for predicted target genes of DE miRNAs. KEGG pathway enrichment for predicted target genes of miRNAs from profile II in the granulosa layer (A) and from profile iv in the theca layer (B). Notably, pathways that involve both ECM-receptor interaction and focal adhesion were identified in both granulosa and theca layers.

Figure S7 Expression levels of protein-coding genes in relation to steroidogenesis (A), lipid regulation (B), and junctional adhesion (C) during follicle development.
