# Supplementary figures and images for: mRNA and miRNA Transcriptome Profiling of Granulosa and Theca Layers From Geese Ovarian Follicles Reveals the Crucial Pathways and Interaction Networks for Regulation of Follicle Selection
Source: Front Genet. 2019 Oct 23;10:988. doi: 10.3389/fgene.2019.00988 (PMC6820619; doi:10.3389/fgene.2019.00988)

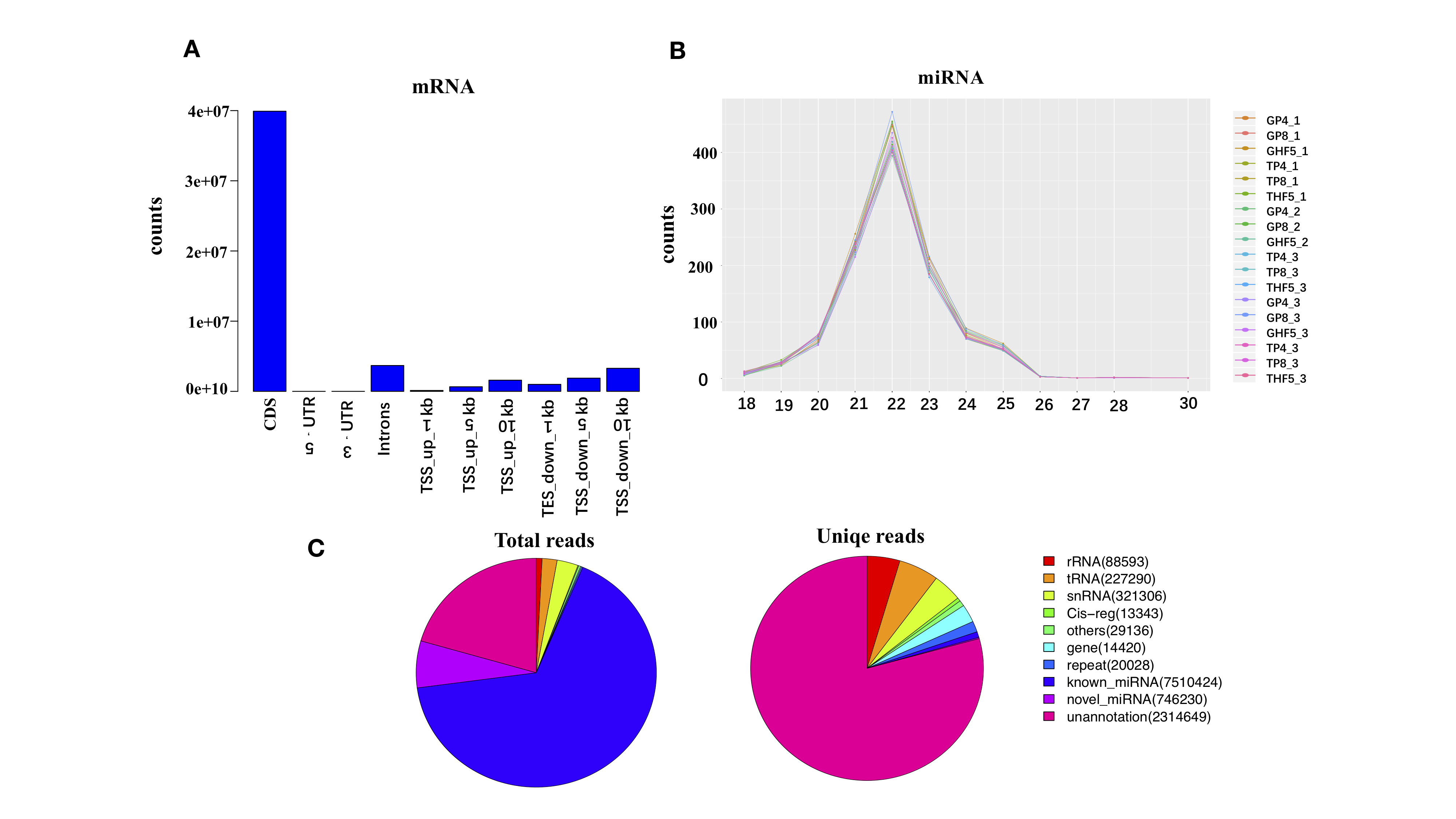

Supplement: Figure S1 — Basic information of RNA-seq and small RNA-seq. (A) Read length distribution of mRNAs; (B) read length distribution of miRNAs; (C) count distribution of total reads (left) and unique small RNAs (right). GP4_1, GP4_2, and GP4_3 represent three replicates of GP4, respectively, and similar definitions were used for GP8, GHF5, TP4, TP8, and THF5. [file Image_1.tiff]

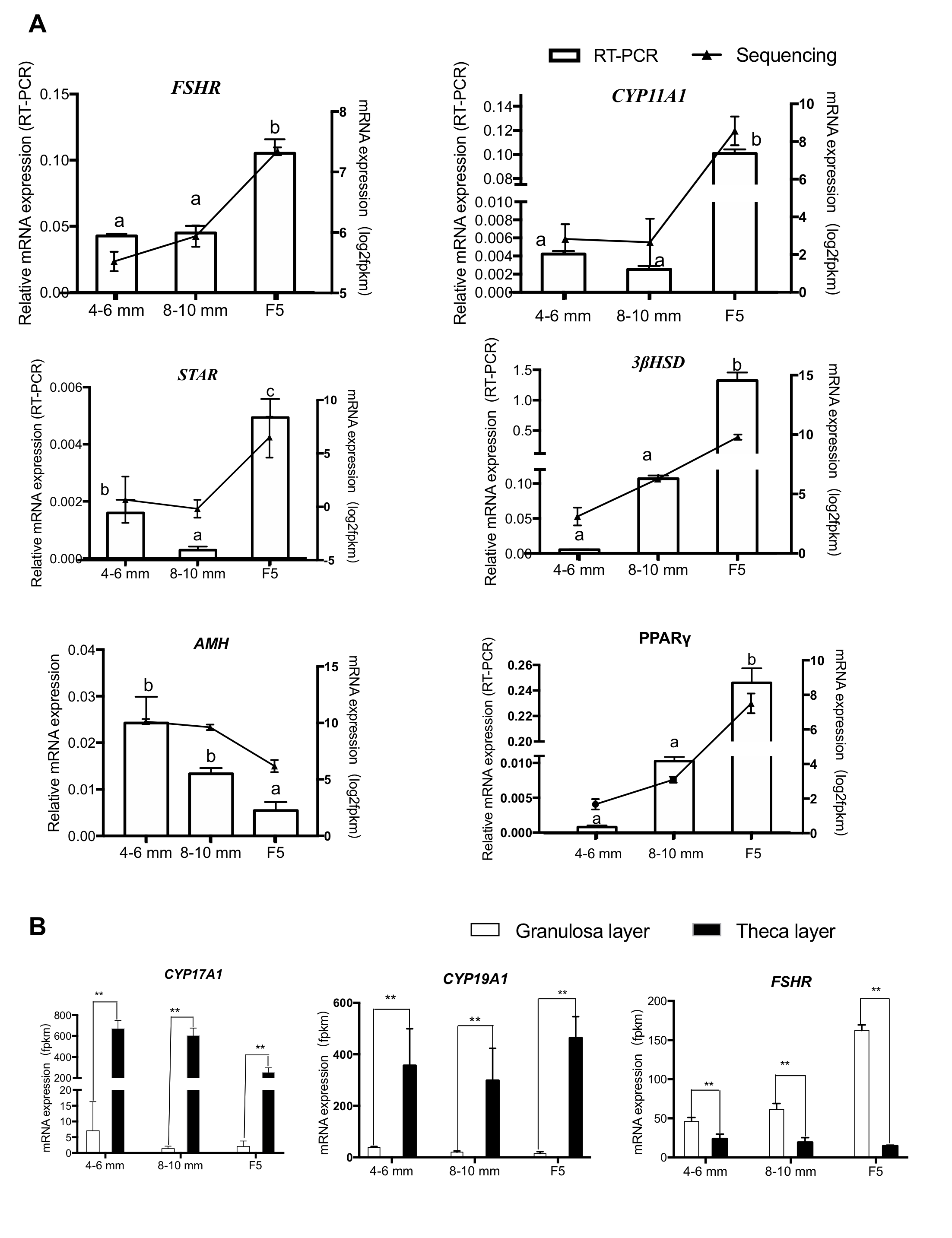

Supplement: Figure S2 — Confirmation of differentially expressed (DE) genes of RNA-seq by qRT-PCR. Mean ± SEM expression of FSHR, CYP11A1, STAR, 3β-HSD, AMH, and PPARγ were quantified in the granulosa layer by RNA-seq and qRT-PCR (A); CYP17A1, CYP19A1, and FSHR were identified in both the granulosa and theca layers by RNA-seq (B) isolated from 4-6-mm (n = 18 ± 3), 8-10-mm (8 ± 2), and F5 (n = 1) follicles. a and b represent significant differences among granulosa layers from three different stages based on the expression of these genes by RT-PCR (Student’s t-test, P < 0.05). ** indicates significant differences between granulosa and theca layer of three different stages (Student’s t-test, P < 0.01). SPSS software was used for statistical analysis. [file Image_2.tiff]

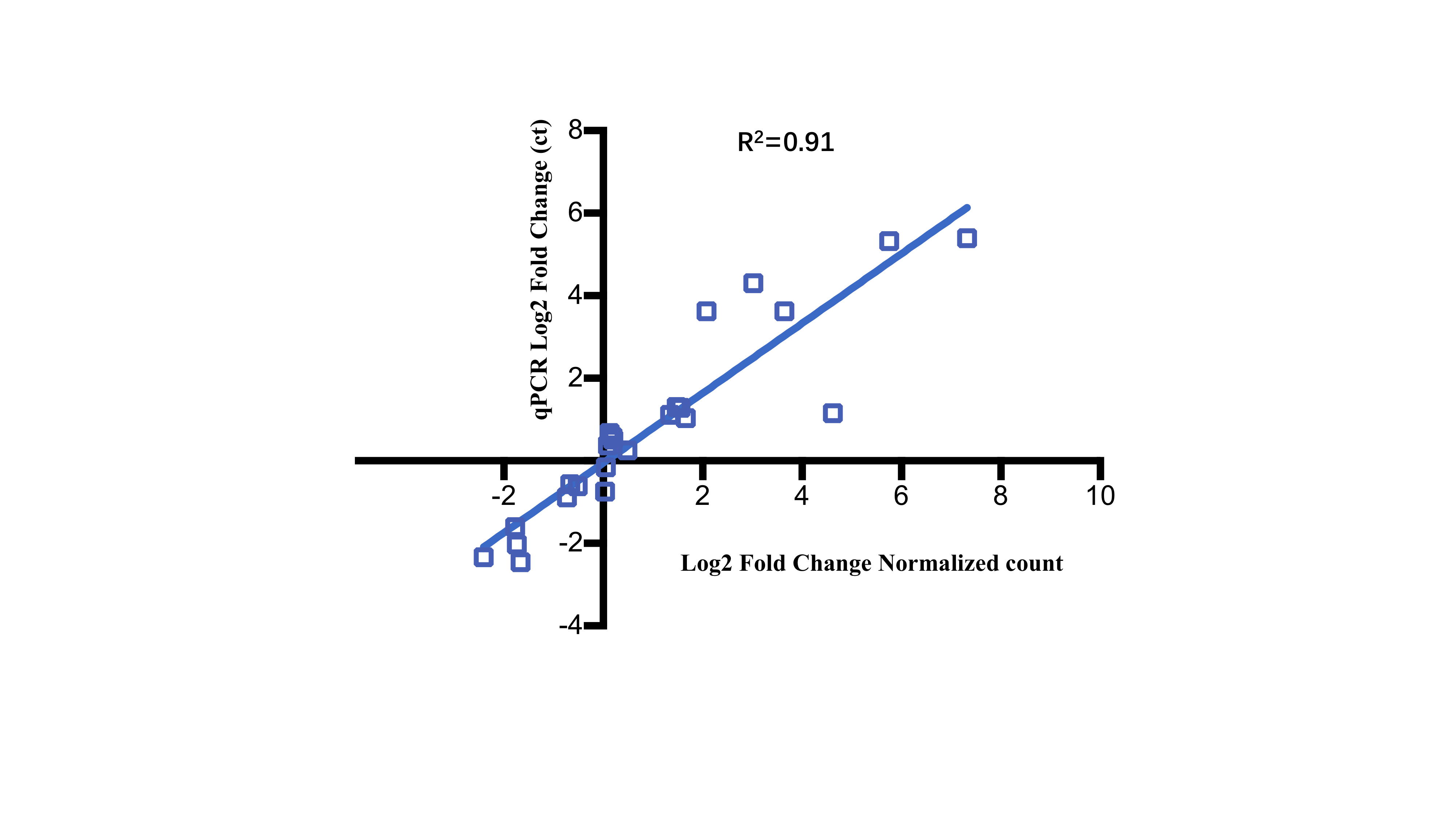

Supplement: Figure S3 — Correlation between the results of RNA-seq and qRT-PCR. [file Image_3.tiff]

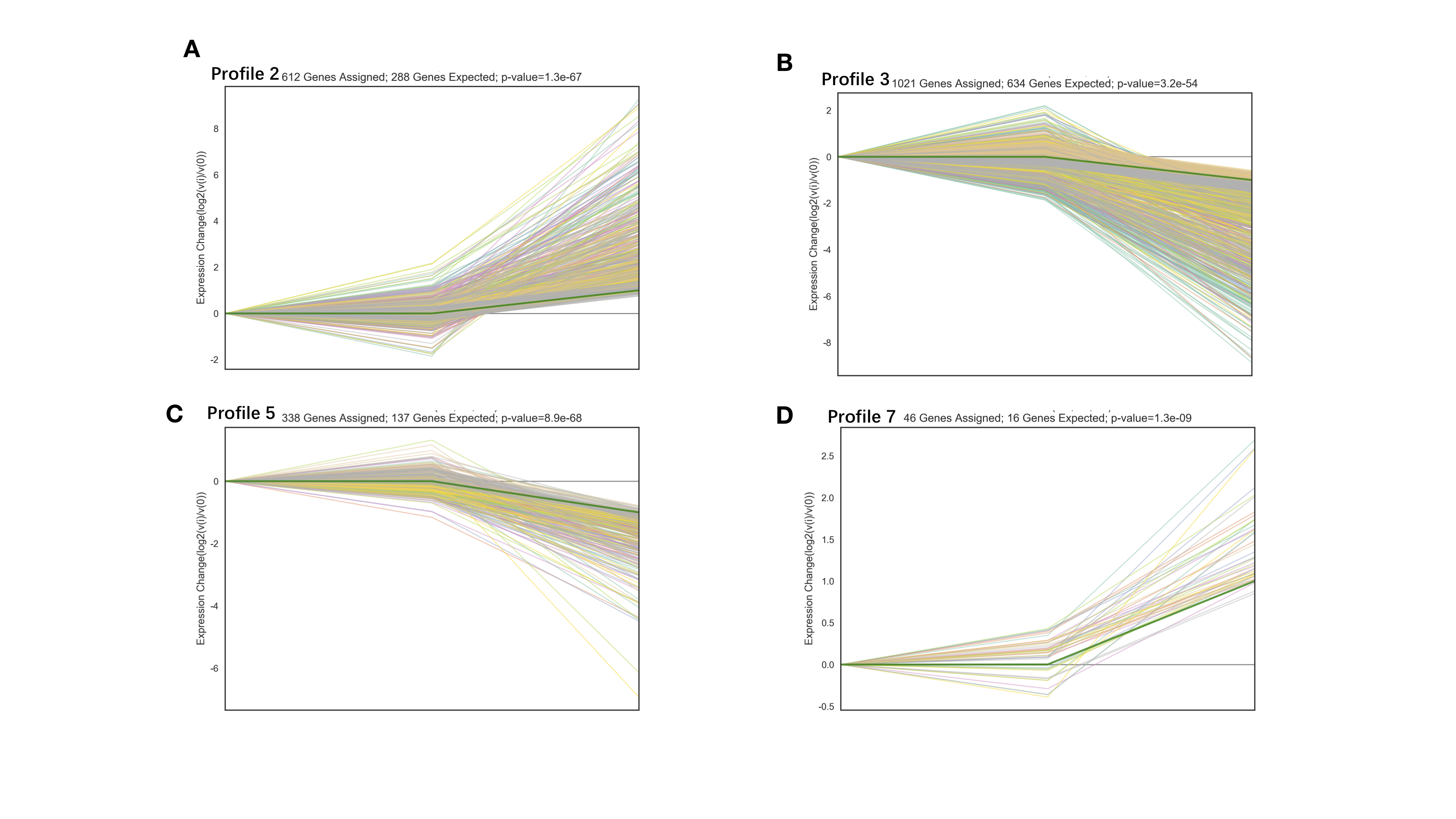

Supplement: Figure S4 — DE mRNAs and miRNAs in the granulosa and theca layers. (A-C): The uniquely up- and down-regulated DE mRNAs between in the GHF5 vs GP8 group and GP8 vs GP4 group (A), and in the THF5 vs TP8 group and TP8 vs TP4 group (C). (B-D): The uniquely up- and down-regulated DE miRNAs between in the GHF5 vs GP8 group and GP8 vs GP4 group (A), and in the THF5 vs TP8 group and TP8 vs TP4 group. [file Image_4.tiff]

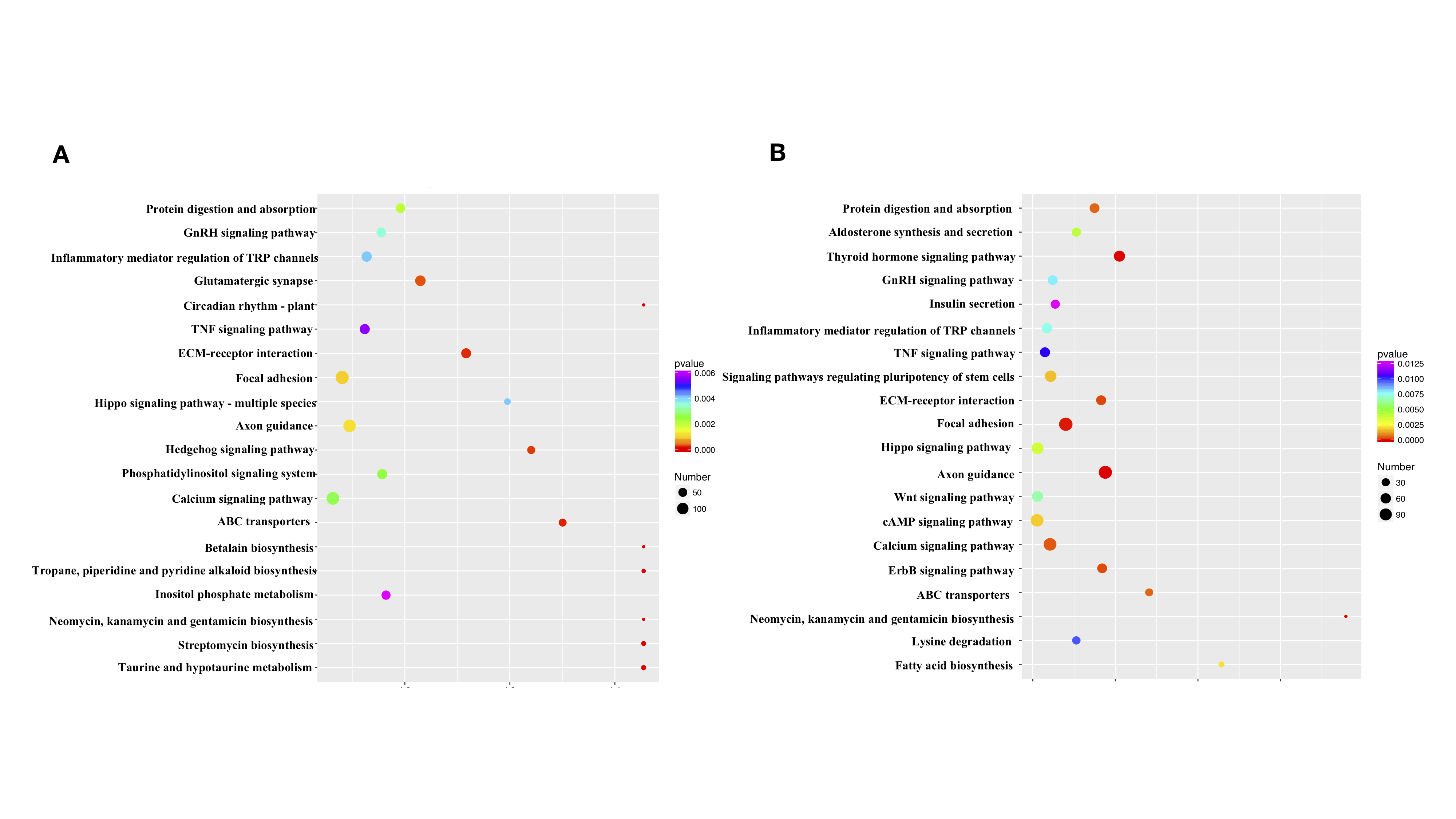

Supplement: Figure S5 — Significant time-series modules of DE mRNAs in the granulosa and theca layers. Four significant time-series clusters were identified, including profiles 2 and 3 in the granulosa layer (A-B), as well as profiles 5 and 7 in the theca layer (C-D). [file Image_5.tiff]

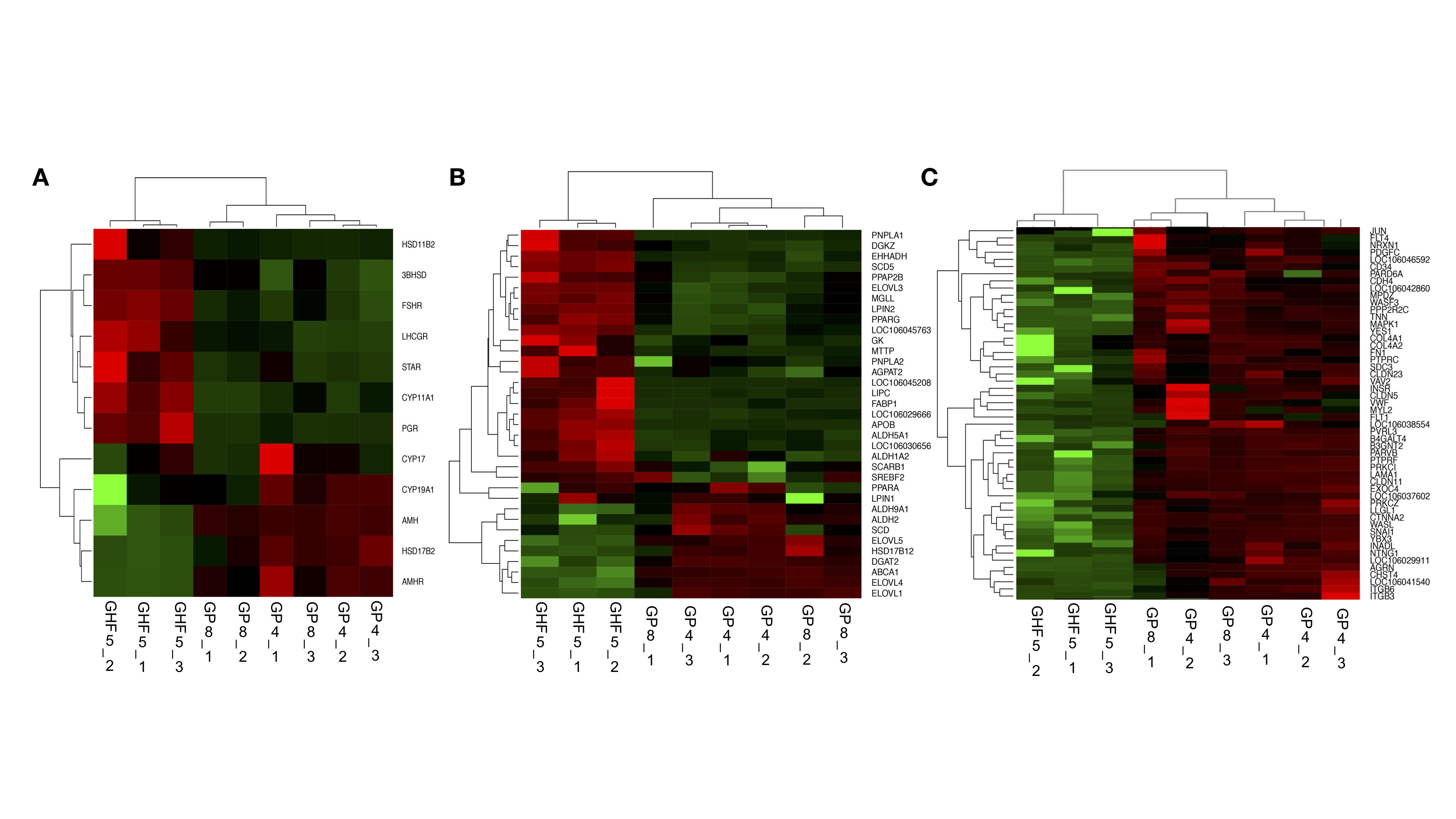

Supplement: Figure S6 — KEGG pathway enrichment for predicted target genes of DE miRNAs. KEGG pathway enrichment for predicted target genes of miRNAs from profile II in the granulosa layer (A) and from profile iv in the theca layer (B). Notably, pathways that involve both ECM-receptor interaction and focal adhesion were identified in both granulosa and theca layers. [file Image_6.tiff]

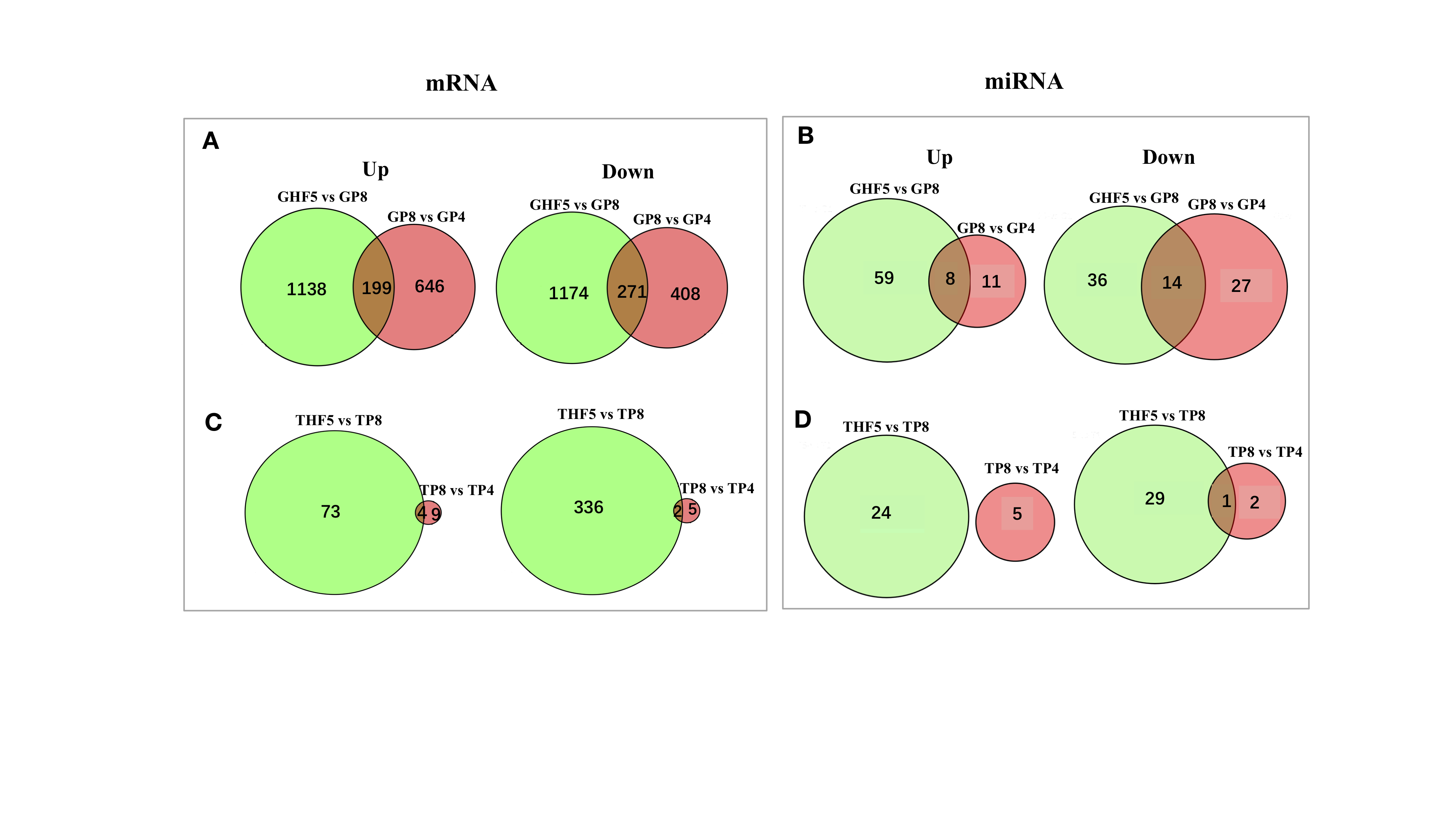

Supplement: Figure S7 — Expression levels of protein-coding genes in relation to steroidogenesis (A), lipid regulation (B), and junctional adhesion (C) during follicle development. [file Image_7.tif]
